# Incidence of invasive salmonella disease in sub-Saharan Africa: a multicentre population-based surveillance study

**DOI:** 10.1016/S2214-109X(17)30022-0

**Published:** 2017-02-11

**Authors:** Florian Marks, Vera von Kalckreuth, Peter Aaby, Yaw Adu-Sarkodie, Muna Ahmed El Tayeb, Mohammad Ali, Abraham Aseffa, Stephen Baker, Holly M Biggs, Morten Bjerregaard-Andersen, Robert F Breiman, James I Campbell, Leonard Cosmas, John A Crump, Ligia Maria Cruz Espinoza, Jessica Fung Deerin, Denise Myriam Dekker, Barry S Fields, Nagla Gasmelseed, Julian T Hertz, Nguyen Van Minh Hoang, Justin Im, Anna Jaeger, Hyon Jin Jeon, Leon Parfait Kabore, Karen H Keddy, Frank Konings, Ralf Krumkamp, Benedikt Ley, Sandra Valborg Løfberg, Jürgen May, Christian G Meyer, Eric D Mintz, Joel M Montgomery, Aissatou Ahmet Niang, Chelsea Nichols, Beatrice Olack, Gi Deok Pak, Ursula Panzner, Jin Kyung Park, Se Eun Park, Henintsoa Rabezanahary, Raphaël Rakotozandrindrainy, Tiana Mirana Raminosoa, Tsiriniaina Jean Luco Razafindrabe, Emmanuel Sampo, Heidi Schütt-Gerowitt, Amy Gassama Sow, Nimako Sarpong, Hye Jin Seo, Arvinda Sooka, Abdramane Bassiahi Soura, Adama Tall, Mekonnen Teferi, Kamala Thriemer, Michelle R Warren, Biruk Yeshitela, John D Clemens, Thomas F Wierzba

**Affiliations:** a International Vaccine Institute, SNU Research Park, Seoul, South Korea; b Bandim Health Project, Bissau, Guinea-Bissau; c Research Center for Vitamins and Vaccines, Copenhagen, Denmark; d Kumasi Centre for Collaborative Research in Tropical Medicine, Kwame Nkrumah University of Science and Technology, Kumasi, Ghana; e Faculty of Medicine, University of Gezira, Wad Medani, Sudan; f Johns Hopkins Bloomberg School of Public Health, Baltimore, MD, USA; g Armauer Hansen Research Institute, Addis Ababa, Ethiopia; h Oxford University Clinical Research Unit, Ho Chi Minh City, Vietnam; i Kilimanjaro Christian Medical Centre, Moshi, Tanzania; j Division of Infectious Diseases and International Health, Duke University Medical Center, Durham, NC, USA; k Centers for Disease Control and Prevention, Nairobi, Kenya; l WHO-Kenya Country Office, Nairobi, Kenya; m Global Health Institute, Emory University, Atlanta, GA, USA; n Duke Global Health Institute, Duke University, Durham, NC, USA; o Centre for International Health, University of Otago, Dunedin, New Zealand; p Bernhard Nocht Institute for Tropical Medicine, Hamburg, Germany; q German Center for Infection Research, Hamburg—Borstel—Lübeck, Germany; r Faculty of Science, University of Hafr Al Batin, Hafr Al Batin, Saudi Arabia; s Schiphra Hospital, Ouagadougou, Burkina Faso; t National Institute for Communicable Diseases, Johannesburg, South Africa; u Faculty of Health Sciences, University of the Witwatersrand, Johannesburg, South Africa; v Global and Tropical Health Division, Menzies School of Health Research, Charles Darwin University, Australia; w Institute of Tropical Medicine, Eberhard-Karls University Tübingen, Tübingen, Germany; x Duy Tan University, Da Nang, Vietnam; y National Center for Emerging and Zoonotic Infectious Diseases, Centers for Disease Control and Prevention, Atlanta, GA, USA; z Institute Pasteur de Dakar, Dakar, Senegal; aa Center for Clinical Research, Kenya Medical Research Institute, Nairobi, Kenya; ab Laboratory of Microbiology, University of Antananarivo, Antananarivo, Madagascar; ac Institut Supérieur des Sciences de la Population, University of Ouagadougou, Ouagadougou, Burkina Faso; ad Schiphra Hospital, Ouagadougou, Burkina Faso; ae Institute of Medical Microbiology, University of Cologne, Cologne, Germany; af University Cheikh Anta Diop de Dakar, Dakar, Senegal; ag International Centre for Diarrheal Disease Research, Bangladesh, Dhaka, Bangladesh; ah University of California Fielding School of Public Health, Los Angeles, CA, USA

## Abstract

**Background:**

Available incidence data for invasive salmonella disease in sub-Saharan Africa are scarce. Standardised, multicountry data are required to better understand the nature and burden of disease in Africa. We aimed to measure the adjusted incidence estimates of typhoid fever and invasive non-typhoidal salmonella (iNTS) disease in sub-Saharan Africa, and the antimicrobial susceptibility profiles of the causative agents.

**Methods:**

We established a systematic, standardised surveillance of blood culture-based febrile illness in 13 African sentinel sites with previous reports of typhoid fever: Burkina Faso (two sites), Ethiopia, Ghana, Guinea-Bissau, Kenya, Madagascar (two sites), Senegal, South Africa, Sudan, and Tanzania (two sites). We used census data and health-care records to define study catchment areas and populations. Eligible participants were either inpatients or outpatients who resided within the catchment area and presented with tympanic (≥38·0°C) or axillary temperature (≥37·5°C). Inpatients with a reported history of fever for 72 h or longer were excluded. We also implemented a health-care utilisation survey in a sample of households randomly selected from each study area to investigate health-seeking behaviour in cases of self-reported fever lasting less than 3 days. Typhoid fever and iNTS disease incidences were corrected for health-care-seeking behaviour and recruitment.

**Findings:**

Between March 1, 2010, and Jan 31, 2014, 135 *Salmonella enterica* serotype Typhi (*S* Typhi) and 94 iNTS isolates were cultured from the blood of 13 431 febrile patients. *Salmonella* spp accounted for 33% or more of all bacterial pathogens at nine sites. The adjusted incidence rate (AIR) of *S* Typhi per 100 000 person-years of observation ranged from 0 (95% CI 0–0) in Sudan to 383 (274–535) at one site in Burkina Faso; the AIR of iNTS ranged from 0 in Sudan, Ethiopia, Madagascar (Isotry site), and South Africa to 237 (178–316) at the second site in Burkina Faso. The AIR of iNTS and typhoid fever in individuals younger than 15 years old was typically higher than in those aged 15 years or older. Multidrug-resistant *S* Typhi was isolated in Ghana, Kenya, and Tanzania (both sites combined), and multidrug-resistant iNTS was isolated in Burkina Faso (both sites combined), Ghana, Kenya, and Guinea-Bissau.

**Interpretation:**

Typhoid fever and iNTS disease are major causes of invasive bacterial febrile illness in the sampled locations, most commonly affecting children in both low and high population density settings. The development of iNTS vaccines and the introduction of *S* Typhi conjugate vaccines should be considered for high-incidence settings, such as those identified in this study.

**Funding:**

Bill & Melinda Gates Foundation.

## Introduction

Salmonella infections contribute substantially to global morbidity and mortality.^1^, ^2^ The best described invasive salmonella serovars are *Salmonella enterica* serotype Typhi (*S* Typhi), causing typhoid fever, and *S enterica* serotype Paratyphi A, B, and C (*S* Paratyphi A, B, and C), which cause paratyphoid fever. Other non-typhoidal salmonella (NTS) serovars that typically cause self-limiting diarrhoea can also cause systemic infections, refered to as invasive NTS (iNTS) disease.^3^ Globally, typhoid fever is estimated to cause 21·7 million illnesses and 217 000 fatalities annually, and iNTS disease is estimated to cause 3·4 million illnesses and 681 000 fatalities annually.^1^, ^2^

Substantial knowledge gaps exist regarding the distribution of typhoid fever and iNTS disease in Africa. The few existing studies,^4^, ^5^, ^6^, ^7^, ^8^ reported over differing time periods and using various protocols, have been extrapolated and contribute to existing typhoid fever estimates, which limits international generalisability. The scarcity of data in sub-Saharan Africa prompted WHO, in 2008, to request more epidemiological information to reliably estimate the incidence of typhoid fever and iNTS disease and the antimicrobial susceptibilities of the corresponding organisms.^9^ Consequently, between 2010, and 2014, we established 13 surveillance sites across sub-Saharan Africa in locations where typhoid fever had been previously reported. This network formed the Typhoid Fever Surveillance in Africa Program (TSAP) and served as a platform to implement standardised surveillance of febrile illness and cross-sectional studies to investigate the health-care-seeking behaviour of the surveyed populations.^10^, ^11^, ^12^ Here, we present the adjusted incidence estimates of typhoid fever and iNTS disease and the antimicrobial susceptibility profiles of the causative agents at the 13 selected surveillance sites.

**Research in context**

**Evidence before this study**

We did a literature search using PubMed with the following search terms: (“typhoid” OR “typhoid fever” OR “Salmonella Typhi” OR “*S* Typhi” OR “salmonella infection” OR “enteric fever” OR “non-typhoidal salmonella” OR “NTS”) AND (“incidence”OR “rate” OR “frequency” OR “prevalence” OR “morbidity” OR “burden” OR “surveillance” OR “epidemiology”). We restricted publication dates from Dec 31, 1995, to July 30, 2016, and no language restrictions were applied. The date of our last search was July 30, 2016.

Salmonella infections are a major cause of global morbidity and mortality; however, substantial knowledge gaps exist with regards to the distribution and incidence of disease caused by *Salmonella enterica* serotype Typhi and invasive non-typhoidal salmonella (iNTS) disease in sub-Saharan Africa.

Before the Typhoid Fever Surveillance in Africa Program (TSAP), estimates of typhoid fever incidence data from Africa were available from four vaccine trials and one population-based study in Kenya. Other estimates of invasive salmonella infections originated from different descriptions of bacteraemia in febrile patients in The Gambia, Malawi, Mozambique, and Kenya. These few, unstandardised, published data are not sufficient for understanding the burden of the disease in sub-Saharan Africa.

In 2008, WHO expressed the necessity for more epidemiological information to estimate the incidence and antimicrobial susceptibility of invasive salmonella disease. Consequently, in January, 2009, the International Vaccine Institute (Seoul, South Korea) and the Kenya Medical Research Institute (Kilifi, Kenya) co-hosted a meeting with five other international institutions and 28 investigators from 14 research sites across sub-Saharan Africa. The purpose of the meeting was to review existing data on invasive salmonella infections in sub-Saharan Africa and surveillance infrastructure from sites, and to discuss the way forward to investigate invasive salmonella in the African region. These 28 investigators and the five international institutions presented their data on invasive bacterial disease, focusing on invasive salmonellosis.

The data indicated the presence of typhoid fever and iNTS disease; however, the studies were not standardised in design, data collection, and laboratory techniques. The meeting concluded that unless standardised methods of data collection and diagnostic procedure were used across countries, and patterns of health-care utilisation were understood and accounted for, the real disease burden of invasive salmonella infections in the region would remain unclear. As a result, a consortium was established and members agreed to form a network of surveillance sites in sub-Saharan Africa in areas with previous reports of cases of typhoid fever.

The TSAP was created to address the knowledge gaps on the incidence and antimicrobial resistance patterns of invasive salmonella infections at different countries with previous reports of typhoid fever cases in sub-Saharan Africa. TSAP created a network of 13 surveillance sites across ten countries, and implemented cross-sectional studies to investigate the health-care-seeking behaviour of the populations under surveillance.

**Added value of this study**

Original data collected in TSAP represent the most comprehensive standardised analysis done in sub-Saharan Africa of the incidence and antimicrobial resistance patterns of invasive salmonella infections. The results describe the incidence estimated, adjusted by health-care-seeking behaviour, and antimicrobial susceptibility of typhoid fever and iNTS diseases from 13 sites in ten sub-Saharan Africa countries. For typhoid fever disease, we estimate that the overall incidence is two to three times higher than a previous estimate (10–100 cases per 100 000 people), and is in some settings similar to data from Asia, where the burden is known to be very high. The data also revealed that children aged 2–14 years bear the greatest burden of the disease. For iNTS disease, the data also reflect a high incidence, especially in young children, and in specific sites (Ghana) the incidence could be more than five times that previously estimated.

**Implications of all the available evidence**

The results of this study underscore the need for preventive measures, including vaccines, improved sanitation and hygiene, malaria control, antiretroviral therapy programmes, and improved nutrition. The results also emphasise that the implementation of effective antimicrobials might be impaired by the presence and potential increase of drug-resistance salmonella strains in the region. The advent of typhoid conjugate vaccines might provide more powerful tools to control typhoid fever; the first vaccine, which was manufactured in India, has already been submitted to WHO for prequalification. Data from this study will be included in the GAVI Alliance review of potential subsidies for typhoid fever vaccines in 2017; their recommendation will be crucial for the deployment of these vaccines. Hence, an urgent need exists to understand the pragmatic aspects of vaccine targeting and delivery, particularly given the burden of disease in children, the associated risk factors, and the focal nature of the disease. Further assessment of the incidence in infants (0–5 months *vs* 6–11 months) and data on severe typhoid fever or iNTS, including mortality, is crucial to determine the potential effect of future vaccines. Our follow-on study—Severe Typhoid in Africa (SETA)—which investigates severe typhoid burden, is underway.

## Methods

### Study design, site selection, and participants

**Figure 1:**
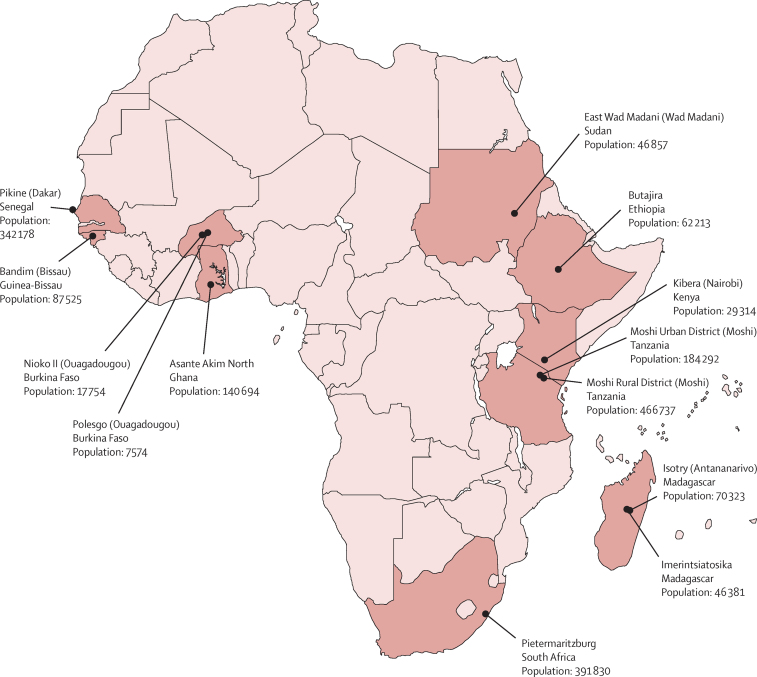
Sites participating in the Typhoid Fever Surveillance in Africa Program

We used a multicentre, population-based, prospective surveillance study design. Selection of the surveillance sites in sub-Saharan Africa was not random; locations were eligible if they had evidence of previous typhoid fever, a laboratory infrastructure suitable for blood culture, an onsite health-care facility, and staff experienced in microbiological laboratory research.^10^ 13 sites in ten countries were selected (figure 1), four of which already had established surveillance systems: Pietermaritzburg, South Africa; Asante Akim North, Ghana; Moshi Urban District and Moshi Rural District, Tanzania; and Kibera, Kenya. Four sites were part of the International Network for the Demographic Evaluation of Populations and Their Health (INDEPTH): Polesgo and Nioko II, Burkina Faso; Butajira, Ethiopia; and Bandim, Guinea-Bissau. These sites had functional Health and Demographic Surveillance Systems (HDSS) in place.^13^ Additional surveillance sites were Isotry and Imerintsiatosika, Madagascar; Pikine, Senegal; and East Wad Medani, Sudan. The surveillance system in Kibera was established before TSAP with an active, population-based surveillance component. Home visits were done once every 2 weeks to screen for febrile patients and encourage visits to the affiliated health-care facility. Active surveillance in Kibera was continued throughout TSAP. All other sites implemented passive surveillance.^10^ The ethics committees of all collaborating institutions and the International Vaccine Institute (Seoul, South Korea) approved the study protocol.

The catchment area for each site was determined through health-care facility records and through accessible administrative and demographic data.^11^ We determined the population of each catchment area using the latest census or the INDEPTH database. We categorised sites as urban, rural, or other using setting classifications at each site. Surveillance was implemented in each study location for a period of at least 12 months and recruitment occurred at primary, secondary, and tertiary health-care facilities.

Recruitment was open to outpatients and inpatients who visited any of the health-care facilities participating in TSAP, who resided within the catchment area and presented with tympanic (≥38·0°C) or axillary temperature (≥37·5°C). Inpatients with a reported history of fever for 72 h or longer were excluded, as were patients with residence outside of the catchment area. Asante Akim North recruited children younger than age 15 years only; other sites recruited patients of all ages. Written informed consent preceded recruitment and clinical appraisal forms were completed for all participants.

### Laboratory procedures

We standardised laboratory, quality control, and blood sample collection procedures across sites.^10^ Blood (5–10 mL for adults; 1–3 mL for children) was inoculated into aerobic blood culture bottles and incubated in an automated blood culture system (BD BACTEC, Becton-Dickinson, USA, or BacT/ALERT, BioMérieux, France), with the exception of Sudan, where manual culturing with daily subculturing for up to 5 days was instituted. Gram staining and bacterial identification were done with standard microbiological techniques.^14^ Quality control of preanalytical processes included time and temperature control measures, during which every blood culture bottle was collected, transported, and placed into the incubator. Quality control of analytical processes included sterility and function control of culture media, controls of biochemical reactions, and antimicrobial susceptibility testing. For the quality control of manual culturing in Sudan, additionally, blood culture bottles were inoculated weekly with a suspension containing *Escherichia coli* or *Staphylococcus aureus* references. Inoculated blood culture bottles were incubated overnight and verified for growth by subculture.

Contaminants were defined as organisms not typically associated with bloodstream infections; these included non-pathogens and those more commonly associated with commensal skin microbiota, including coagulase-negative *Staphylococci, Bacillus* spp, and *Micrococcus* spp. Antimicrobial susceptibility testing was done by disc diffusion according to Clinical and Laboratory Standards Institute^15^ standards for ampicillin, amoxicillin-clavulanic acid, chloramphenicol, co-trimoxazole, ceftriaxone, and ciprofloxacin. Multidrug resistance was defined as resistance to ampicillin or amoxicillin-clavulanic acid, chloramphenicol, and co-trimoxazole. Isolates with intermediate susceptibility were classified as resistant. Malaria blood smears were routinely done, except in South Africa. In Ethiopia, rapid diagnostic tests (SD BIOLINE Malaria Ag *Pf*/*Pv*, SD Standard Diagnostics, Yongin, South Korea) were used in addition to routine malaria blood smears.

### Health-care utilisation survey and person-years of observation calculation

The health-care-seeking behaviour of the populations under surveillance was investigated with the assumption that access to the TSAP health-care facility was non-uniform throughout the population.^16^, ^17^ A standardised and pretested health-care utilisation survey was implemented in a representative sample of households randomly selected from each study area.^11^ We investigated health-care-seeking behaviour in cases of self-reported fever lasting less than 3 days. The first choice of health-care facility in cases of fever was categorised by age-stratified groups and used to calculate the proportion of individuals from the catchment population who visited this TSAP health-care facility. This proportion constituted an adjustment factor to correct incidences. The time at risk in person-years of observation (PYO) stratified by age was calculated using the adjusted population. In HDSS sites, each resident contributed to PYO for the time present in the study area during the recruitment period. In non-HDSS sites, we calculated PYO by projecting the catchment population from the start to the end of the study recruitment period, and multiplied the calculated average population by the number of years of surveillance duration.

### Statistical analysis

We established a multicountry database using FoxPro software. We excluded patients from the analysis who were recruited during pilot testing, failed to meet inclusion criteria, or had incomplete laboratory results. We estimated incidences per 100 000 PYO. Confirmed invasive salmonella cases, stratified by age group (0–1 years, 2–4 years, 5–14 years, and ≥15 years), were adjusted by the specific age-group recruitment proportion. We calculated this proportion by dividing the number of patients with complete data (numerator) by the total number of patients in the study area who had been diagnosed with a febrile illness at a recruitment facility during the surveillance period (denominator). We used health-care facility records, reviewed at the end of the surveillance activities, to estimate the number of patients diagnosed with a febrile illness. The catchment population in PYO, adjusted by health-care-seeking behaviour, was used as the denominator in crude and adjusted incidence rates (AIR).

The 95% CI for AIR was derived on the log-scale and exponentiated. We used the error factor (exp[1·96/√adjusted cases]) to calculate the lower (adjusted rate/error factor) and upper (adjusted rate × error factor) 95% CIs. At the sites in Senegal, Ethiopia, and South Africa, incomplete health-care facility records did not allow for the estimation of the recruitment proportion and calculation of AIRs; for these sites we present crude rates. AIRs for typhoid fever and iNTS were assessed for all other sites. Differences in proportions of blood cultures positive for a pathogen between study years were assessed with the χ^2^ test (SAS, version 9.3).

### Role of the funding source

The funder of the study had no role in study design, data collection, data analysis, data interpretation, or writing of the report. The corresponding author had full access to all the data in the study and had final responsibility for the decision to submit for publication.

## Results

**Figure 2:**
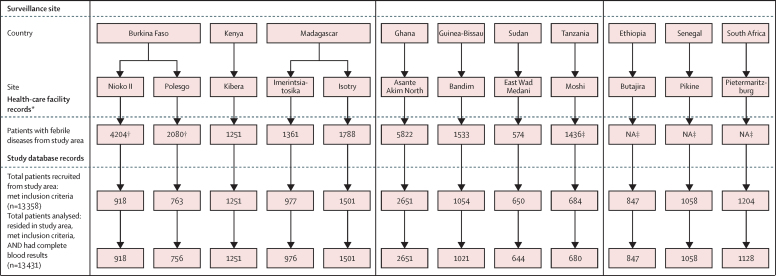
Visits to health-care facilities and recruitment of patients during surveillance period at each site NA=not available. *Data on health facility visits were collected retrospectively, after completion of surveillance period. Diagnosis of febrile illnesses was used at sites when temperature of patients was not recorded. †Number estimated by the proportion of the population under demographic surveillance at each respective site. ‡In Tanzania, before Nov 11, 2011, every fifth eligible patient was recruited; from Nov 11, 2011, every second eligible patient was recruited. This recruitment pattern was applied to this number.

Between March 1, 2010, and Jan 31, 2014, we recruited 13 558 patients from 13 sites who met the inclusion criteria and resided in the catchment areas (Figure 1, Figure 2). We excluded data from 127 (1%) patients because of incomplete laboratory results; data from 13 431 patients were analysed, and 8582 patients (64%) were younger than 15 years (table 1). All patients had one blood culture sample analysed at recruitment and 11 421 (85%) were screened for malaria parasites (table 1). The proportion of contaminated blood cultures ranged from less than 1% in Imerintsiatosika to 24% in Nioko II. The proportion of blood cultures that yielded non-contaminant bacteria varied between sites, ranging from 1% in Imerintsiatosika to 9% in Kibera (table 1). In total, 568 non-contaminant bacteria were isolated from blood samples of febrile patients. The most frequent non-contaminant bacteria isolated were *S* Typhi (135 [24%]), NTS (94 [17%]), *S aureus* (70 [12%]), *E coli* (47 [8%]), and *Streptococcus pneumoniae* (43 [8%]). Of the sites with at least 2 years of surveillance (Asante Akim North, Kibera, and Pietermaritzburg), the proportion of blood cultures that were pathogen positive differed significantly between study years in Kibera only (12% at year 1 and 5% at year 2; p<0·0001; χ^2^ test).

With the exception of East Wad Medani, *Salmonella* spp were isolated from the blood of febrile patients at all sites (135 *S* Typhi and 94 iNTS isolates), which accounted for 33% or more of all isolated bacteria in all but four sites (East Wad Medani, Pietermaritzburg, Butajira, and Isotry). Seasonal variation was not observed at any site (data not shown). The most common iNTS serovars were *S enterica* serotype Typhimurium (38 [40%] of 94), *S enterica* serotype Enteriditis (11 [12%] of 94), and *S enterica* serotype Dublin (10 [11%] of 94). The highest AIRs for typhoid fever in the 15 years or younger age group were observed in Polesgo, Kibera, and Asante Akim North (table 2). *S* Paratyphi A (three isolates) was isolated in Senegal only.

Among age groups of children younger than 15 years, the highest AIR for typhoid fever was observed in children aged 2–4 years from Polesgo, Asante Akim North, Moshi Urban District, and Kibera, and in children aged 5–14 years from Kibera and Polesgo (table 2). The AIR for typhoid fever in adults (aged ≥15 years) was less than 70 per 100 000 PYO at all sites except Moshi Urban District, Kibera, and Polesgo (table 2).

iNTS organisms were more frequently isolated from infants (0–1 years) or children aged 2–4 years than from adults (table 2), except for the sites in Pikine, Moshi Rural District, and Kibera. The AIR for iNTS among children aged 2–4 years was highest in Nioko II, Polesgo, and Asante Akim North. The AIR for iNTS in children younger than 15 years was less than 100 per 100 000 PYO in Kibera, Imerintsiatosika, and in both sites in Tanzania. No iNTS was isolated from sites in Sudan, South Africa, Ethiopia, and Isotry.

The antimicrobial susceptibility profiles of *S* Typhi and iNTS isolates differed between sites (table 3). Overall, 47% of *S* Typhi isolates and 48% of iNTS isolates were multidrug resistant. Most multidrug-resistant *S* Typhi isolates were obtained at the sites in Kenya, Ghana, and Tanzania (both sites combined). Multidrug-resistant iNTS isolates were isolated at the sites in Burkina Faso (both combined), Ghana, Guinea-Bissau, and Kenya (table 3). *S* Typhi isolates that had reduced ciprofloxacin susceptibility were cultured in Kenya and South Africa, only; one ciprofloxacin-resistant *S* Paratyphi A organism was isolated in Senegal. Ciprofloxacin-resistant iNTS was similarly uncommon, isolated only in Burkino Faso (once at the Nioko II site) and in Ghana. One iNTS isolate in Kenya was resistant to ceftriaxone (table 3).

## Discussion

This study identified *Salmonella* as a major cause of invasive bacterial febrile illness across sub-Saharan Africa, affecting children aged 2–14 years rather than adults, and arising in both high-population and low-population density settings. Other major causes of invasive bacterial febrile illnesses varied by country; *E coli* and *S aureus* were the most frequent non-*Salmonella* pathogens isolated from blood.

Results from previous studies^18^, ^19^ suggest that typhoid fever in some sub-Saharan Africa settings occurs predominately in urban settlements with high-population densities, and that disease incidence could have been overestimated by the use of the Widal test. Our study, done using a standardised protocol in both urban and rural settings, indicated high incidences of typhoid fever and iNTS in areas with high-population and low-population densities. Separate analyses done at the Ghana site confirmed this observation and revealed a higher disease incidence in children living in rural areas than in those living in urban areas.^20^ Furthermore, we observed variable incidences of typhoid fever and iNTS among neighbouring populations in Burkina Faso, and in the same populations in Kenya and Ghana in consecutive years, indicating a focal nature and a fluctuating burden of iNTS disease.

A previous global estimate of the burden of typhoid fever indicated that south-central and east-central Asia had the highest incidences of typhoid fever with more than 100 cases per 100 000 people annually; Africa was estimated to have a medium incidence (10–100 cases per 100 000).^1^ The AIR for typhoid fever estimated in our study reveals a higher burden than previously estimated.^1^ Four sites had an overall AIR for typhoid fever of more than 100 per 100 000 PYO, five sites had an AIR for typhoid fever of more than 100 per 100 000 PYO in children younger than 15 years, and six sites had an AIR for typhoid fever of more than 100 per 100 000 PYO in at least one age group. Similar to the Diseases of the Most Impoverished programme done in Asia,^21^ our results show that children aged 2–14 years bear the greatest burden of typhoid fever. Notably, our data indicate that the AIR for typhoid fever at TSAP sites was equal to or even greater than incidences reported in five Asian countries in the early 2000s.^21^, ^22^

For iNTS disease, we observed an AIR equal or higher than previously estimated and a bimodal age distribution with very young children and adults being the key age group for symptomatic infection.^2^ This age distribution differed from that observed for typhoid fever, in which children aged 2–14 years were the most affected, and emphasises substantial differences in the epidemiology of typhoid fever and iNTS disease. Malaria, malnutrition, and HIV infections have been reported to be associated with iNTS disease in Africa.^23^ At TSAP sites, a higher AIR for iNTS was observed in children with a malaria positivity rate of 30% or more than in those with a lower positivity rate; this observation was confirmed in a separate analysis.^24^

Results of our study identified a high prevalence of resistance against first-line antimicrobials in both *S* Typhi and iNTS infections. Reduced susceptibility to ciprofloxacin was identified in *S* Typhi from Kibera and Pietermaritzburg. Multidrug-resistant iNTS isolates were isolated at several sites and have been isolated in sub-Saharan Africa previously.^18^, ^25^, ^26^ Furthermore, a single iNTS isolate from Kibera showed resistance to ceftriaxone. Genomic analyses^27^ have described the spread of *S* Typhi haplotype H58 into Africa, a multidrug-resistant strain associated with reduced ciprofloxacin susceptibility. The susceptibility patterns observed in our study are concerning, particularly because some antimicrobial-resistant *S* Typhi can have a selective fitness advantage.^28^ Concerted measures are needed to monitor the emergence of fluoroquinolone-resistant *Salmonella*.^29^, ^30^, ^31^, ^32^

We made all efforts to minimise bias; however, our study has some limitations. First, we did not adjust the disease incidences for blood culture sensitivity, which is approximately 40–60% of bone marrow culture.^33^, ^34^, ^35^, ^36^, ^37^ This correction factor is inconsistently applied in studies and, if applied here, the incidences presented would double. The restricted sensitivity of blood culture to detect *Salmonella* pathogens applies to other bacterial pathogens as well—ie, *S pneumoniae* and *Haemophilus influenzae* type b—however, those are universally recognised as important infections for which vaccines are cost-effective, and vaccination programmes have been established. Second, our results represent incidence in sites selected because of their previous reports on typhoid fever. The site selection strategy limits the generalisability of the AIR to other locations and might result in the reduced detection of iNTS disease. Third, given the vast number of patients (and restricted diagnostics capacity), not every patient with a history of fever was enrolled—eg, at sites where inpatients were recruited, patients with a fever for 72 h or longer were excluded to minimise the inclusion of patients pretreated with antimicrobials and to maximise blood culture yield. Fourth, the proportion of the catchment population using the TSAP health-care facilities for febrile illness was low in some sites, and antimicrobial treatment before blood collection and its potential effect on blood culture sensitivity were not assessed. Fifth, the classification of the settings as either urban, rural, semi-urban, or urban-slum reflects the classification commonly used at each site and does not refer to a standard definition; instead, the population density of each site is presented to make setting comparisons. Sixth, sites with no previous experience of blood collection for blood culture assessment had a higher incidence of contamination than sites with previous experience of blood collection (South Africa, Ghana, Tanzania, and Kenya); these incidences might have led to errors in clinical interpretation and uncertainty to distinguish between clinically significant bacteraemia and contamination. Available isolates and blood samples collected from participants were PCR tested at the reference lab to minimise misclassification of isolated organisms. Seventh, the site in Ghana recruited only children younger than 15 years and the proportion of recruited inpatients varied greatly across all sites. Finally, data on disease severity, complications, mortality, and HIV status were not assessed because these were not primary study objectives. Despite these limitations, this multisite study, the largest study of typhoid fever and iNTS done across sub-Saharan Africa to date, provides the most current and accurate incidence figures for these major infectious diseases across the continent and has substantial implications for their control.

We surmise that the incidence of invasive salmonella infections among children in sub-Saharan Africa is much higher than previously estimated, underscoring the need for preventive measures. Therefore, until access to safe drinking water and improved sanitation is greatly expanded, the prevention of typhoid fever will require immunisation and effective treatment options.^38^ The advent of new typhoid fever conjugate vaccines might provide more powerful tools for disease control; the first typhoid fever conjugate vaccine (Bharat Biotech, Hyderabad) has been submitted to WHO for prequalification. Data from TSAP will be incorporated into the GAVI Alliances' review of potential subsidies for typhoid fever vaccines in 2017; their recommendation will be crucial for deployment of these vaccines. Hence, the need to understand the pragmatic aspects of vaccine targeting and delivery is pressing, particularly given the burden of disease in children, the associated risk factors, and the focal and unpredictable nature of the disease. Similarly, in the absence of vaccines targeting iNTS disease, prevention will require a major investment in infrastructure for diagnosis and effective treatment of iNTS disease. When appropriate diagnosis and treatment are available, the use of effective antimicrobials might be impaired by the presence and potential increase of multidrug-resistant salmonella. Further assessment of incidences in infants (0–5 months *vs* 6–11 months) and data on severe typhoid fever or iNTS, including mortality, is crucial to determine the potential effect of future vaccines. We are currently undertaking a follow-on study—Severe Typhoid in Africa (SETA)—which investigates severe typhoid burden.

We conclude that typhoid fever and iNTS disease are major agents of invasive bloodstream infections in urban and rural locations, affecting children more commonly than adults across sub-Saharan Africa. Immunisation of high-risk age groups with existing and new vaccines should be a priority. The next generation of epidemiological studies in sub-Saharan Africa needs to provide better data regarding the severity and mortality of typhoid fever and iNTS to guide the introduction of new typhoid and iNTS vaccines. Lastly, the accelerated development and introduction of iNTS vaccines needs to become a fundamental goal on the global health agenda.

For the **study protocol** see http://www.ivi.int/?page_id=12479&uid=922&mod=document

## Figures and Tables

**Table 1:** Demographics and laboratory results of the sites in the Typhoid Fever Surveillance in Africa Program

	**Nioko II, Burkina Faso**	**Polesgo, Burkina Faso**	**Bandim, Guinea-Bissau**	**Pikine, Senegal**	**Asante Akim North, Ghana**	**East Wad Medani, Sudan**	**Butajira, Ethiopia**	**Imerintsiatosika, Madagascar**	**Isotry, Madagascar**	**Pietermaritzburg, South Africa**	**Moshi Urban District, Tanzania**	**Moshi Rural District, Tanzania**	Kibera, Kenya*
**Surveillance sites**													
Type of health-care facility (IPD, OPD)	1 hospital (IPD, OPD)	1 health-care centre (OPD)	1 hospital, 1 health-care centre (IPD, OPD)	1 hospital, 3 health-care centres (IPD, OPD)	1 hospital (IPD)	3 health-care centres (OPD)	1 hospital, 3 health-care centres (IPD, OPD)	1 health-care centre (OPD)	1 health-care centre (OPD)	1 hospital (IPD)	1 hospital (IPD, OPD)	1 hospital (IPD, OPD)	1 health-care centre (OPD)
Setting†	Semi-urban	Semi-urban	Urban	Urban and urban slum	Urban and rural	Urban	Semi-urban and rural	Rural	Urban	Urban	Urban	Rural	Urban slum
Population density, people per km^2^	2204	5163	17 078	16 695	121	7209	6545	225	29 301	1191	3069	332	77 000
Surveillance period (months)‡	April, 2012, to September, 2013 (18)	April, 2012, to September, 2013 (18)	December, 2011, to April, 2013 (17)	December, 2011, to April, 2013 (17)	March, 2010, to May, 2012 (27)	July, 2012, to July, 2013 (13)	May, 2012, to January, 2014 (21)	November, 2011, to June, 2013 (20)	February, 2012, to May, 2013 (16)	February, 2012, to January, 2014 (24)	September, 2011, to May, 2013 (21)	September, 2011, to May, 2013 (21)	January, 2012, to December, 2013 (24)
Source of catchment population	HDSS 2011§	HDSS 2011§	HDSS 2011§	Ministry of Health 2012¶	Census 2010‖	Census 2008**	HDSS 2012§	Ministry of Health 2010¶	Ministry of Health 2010¶	Census 2010††	Census 2012‡‡	Census 2012^f^	KEMRI/CDC 2012^g^
Collaborating research institution	UoO	UoO	BHP	IPD§	KCCR/BNITM	UoG	AHRI	UoA	UoA	NICD	KCMC/Duke	KCMC/Duke	KEMRI/US-CDC
**Patient demographics**													
Patients analysed, N§§	918	756	1021	1058	2651	644	847	976	1501	1128	406	274	1251
Median age, years (IQR)	4 (1–12)	7 (3–21)	3 (1–7)	22 (14–32)	2 (0–5)	15 (9–32)	11 (5–25)	20 (9–32)	26 (17–40)	3 (1–29)	7 (1–29)	19 (2–39)	7 (4–14)
0–1 years, n (% of N)	247 (27%)	117 (15%)	369 (36%)	9 (1%)	1114 (42%)	2 (<1%)	74 (9%)	66 (7%)	12 (1%)	427 (38%)	114 (28%)	67 (24%)	99 (8%)
2–4 years, n (% of N)	235 (26%)	148 (20%)	271 (27%)	23 (2%)	841 (32%)	41 (6%)	124 (15%)	87 (9%)	58 (4%)	209 (19%)	62 (15%)	37 (14%)	312 (25%)
5–14 years, n (% of N)	228 (25%)	252 (33%)	274 (27%)	255 (24%)	696 (26%)	275 (43%)	303 (36%)	184 (19%)	234 (16%)	95 (8%)	56 (14%)	26 (9%)	539 (43%)
≥15 years, n (% of N)	208 (23%)	239 (32%)	107 (10%)	771 (73%)	NA	326 (51%)	346 (41%)	639 (65%)	1197 (80%)	397 (35%)	174 (43%)	144 (53%)	301 (24%)
Female patients, n (% of N)	467 (51%)	404 (53%)	487 (48%)	468 (44%)	1204 (45%)	348 (54%)	433 (51%)	570 (58%)	997 (66%)	586 (52%)	211 (52%)	149 (54%)	622 (50%)
Inpatients, n (% of N)	66 (7%)	NA¶¶	224 (22%)	241 (23%)	2651 (100%)	NA¶¶	31 (4%)	NA¶¶	NA¶¶	1128 (100%)	220 (54%)	156 (57%)	NA¶¶
**Laboratory results**													
Total blood culture, N	918	756	1021	1058	2651	644	847	976	1501	1128	406	274	1251
Total contaminated blood cultures, n (% of N)	220 (24%)	145 (19)	125 (12%)	96 (9%)	182 (7%)	54 (8%)	90 (11%)	6 (1%)	49 (3%)	192 (17%)	8 (2%)	13 (5%)	16 (1%)
Total positive blood cultures, n (% of N)‖‖	29 (3%)	31 (4)	30 (3%)	31 (3%)	175 (7%)	16 (2%)	26 (3%)	11 (1%)	30 (2%)	51 (5%)	17 (4%)	11 (4%)	110 (9%)
Positive for malaria, n (% of all patients tested)***	430/908 (47%)	444/744(60%)	206/525(39%)	297/1058(28%)	1139/2651(43%)	254/632(40%)	110/822(13%)	19/955(2%)	2/274(1%)	0	4/406(1%)	2/274 (1%)	226/956(24%)

UoO=University of Ouagadougou, Ouagadougou. BHP=Bandim Health Project, Bissau. IPD=Institute Pasteur de Dakar, Dakar. KCCR/BNITM=Kumasi Centre for Collaborative Research in Tropical Medicine, Kumasi/Bernhard Nocht Institute for Tropical Medicine, Hamburg, Germany. UoG=University of Gezira, Wad Medani. AHRI=Armauer Hansen Research Institute, Addis Ababa. UoA=University of Antananarivo, Antananarivo. NICD=National Institute for Communicable Diseases, Johannesburg. KCMC/Duke=Kilimanjaro Christian Medical Center, Moshi/Duke University Medical Center, Durham, NC, USA. KEMRI/US-CDC=Kenya Medical Research Institute/US Centers for Disease Control and Prevention, Nairobi. IPD=inpatient department. OPD=outpatient department. HDSS=Health and Demographic Surveillance System. KEMRI=Kenya Medical Research Institute. NA=not available.

* In Kibera, active population mobilisation was done in addition to passive surveillance.

† Setting reflects the classification commonly used at each site and does not refer to a standard definition.

‡ Surveillance activities were scheduled for 12 months in Burkina Faso, Guinea-Bissau, Senegal, Sudan, Ethiopia, and Madagascar and for 24 months in Ghana, Kenya, South Africa, and Tanzania. If funds allowed, the scheduled period was extended.

§ Population data were provided from the HDSS country office.

¶ Population data for Senegal and Madagascar were provided by Ministry of Health. Population data correspond to the 2012 population census and 2010 estimated population for the area, respectively.

‖ Population data for Ghana were obtained from the Ghana Statistical Service, 2010 population, and housing census. It includes 53 towns distributed in what is now Asante Akim North and Central.

** Population data for Sudan were provided by the Statistics Department, Population Center, University of Gezira, Sudan, and correspond to year 2008.

†† Population data for South Africa were provided by the Statistics Department in South Africa and corresponds to the 2011 census.

‡‡ Population data for Tanzania were provided by the National Bureau of Statistics and correspond to the 2012 population and housing census.

§§ Patients who met inclusion criteria, consented to take part in the study, and had a blood culture taken and a documented blood culture result.

¶¶ Recruitment health-care facility providing outpatient services only.

‖‖ Positive for non-contaminant isolates.

*** Denominator differs from all blood cultures analysed because of missing values. Malaria results are based on blood smears, except for the site in Butajira (52% of patients positive for malaria were diagnosed with malaria rapid tests).

**Table 2:** Invasive salmonella infections across sites in the Typhoid Fever Surveillance in Africa Program

	**Proportion of individuals from study population visiting recruitment facility in case of fever (95% CI)**	**PYO estimation**			**Recruitment proportion**	***Salmonella* Typhi**				**iNTS**			
		Study population	Study population adjusted by health-seeking behaviour	PYO		Crude cases	Crude incidence per 100 000 PYO	Cases adjusted for recruitment	Adjusted incidence per 100 000 PYO (95% CI)	Crude cases	Crude incidence per 100 000 PYO	Cases adjusted for recruitment	Adjusted incidence per 100 000 PYO (95% CI)
**Nioko II, Burkina Faso**													
0–1 years	81% (74–88)	2208	1788	2097	247/1297 (19%)	0	0	0·0	0 (0–0)	3	143	15·8	753 (460–1233)
2–4 years	81% (75–86)	1823	1477	2097	235/1259 (19%)	1	48	5·3	251 (107–590)	3	143	16·0	753 (460–1233)
5–14 years	81% (78–84)	4295	3479	4889	228/889 (26%)	4	82	15·4	315 (191–519)	3	61	12·0	236 (133–420)
<15 years	NA	8326	6744	9083	NA	5	55	20·6	227 (148–350)	9	99	43·1	475 (352–640)
≥15 years	81% (79–83)	9428	7637	10 676	208/759 (27%)	0	0	0·0	0 (0–0)	1	9	4·0	35 (13–96)
All	..	17 754	14 381	19 759	NA	5	25	20·6	104 (68–161)	10	51	46·8	237 (178–316)
**Polesgo, Burkina Faso**													
0–1 years	92% (86–99)	896	824	929	117/475 (25%	0	0	0·0	0 (0–0)	1	108	4·0	431 (162–1147)
2–4 years	83% (76–89)	856	710	992	148/466 (32%)	6	605	18·8	1890 (1202–2972)	2	202	6·0	630 (288–1380)
5–14 years	87% (83–91)	1734	1509	2104	252/510 (49%)	5	238	10·2	485 (263–896)	0	0	0·0	0 (0–0)
<15 years	NA	3486	3043	4025	NA	11	273	29·0	719 (500–1035)	3	75	10·3	255 (138–470)
≥15 years	87% (84–89)	4088	3557	4917	239/629 (38%)	2	41	5·3	107 (46–252)	1	20	3·0	54 (16–179)
All	NA	7574	6600	8942	NA	13	145	34·2	383 (274–535)	4	45	12·9	144 (83–249)
**Bandim, Guinea-Bissau**													
0–1 years	46% (39–54)	10 852	4992	5198	206/631 (33%)	0	0	0·0	0 (0–0)	5	96	15·2	291 (176–482)
2–4 years	43% (37–48)	7307	3142	3866	175/359 (49%)	1	26	2·0	53 (13–208)	1	26	2·0	53 (13–208)
5–14 years	42% (41–48)	19 905	8360	11 101	187/380 (49%)	1	9	2·0	18 (5–72)	2	18	4·0	53 (14–97)
<15 years	NA	38 064	16 494	20 165	NA	2	10	4·1	20 (8–53)	8	40	21·3	116 (69–161)
≥15 years	45% (43–47)	62 694	28 212	37 109	105/163 (64%)	1	3	1·6	4 (1–20)	0	0	0·0	0 (0–0)
All	NA	100 758	44 706	57 274	NA	3	5	5·6	10 (4–22)	8	14	21·3	37 (24–57)
**Asante Akim North, Ghana**													
0–1 years	16% (14–18)	11 222	1760	4080	41%*	2	49	4·9	120 (49–290)	29	711	70·7	1733 (1373–2188)
2–4 years	16% (13–18)	8086	1268	2940	41%*	13	442	31·7	1079 (762–1528)	23	782	56·1	1908 (1469–2479)
5–14 years	16% (15–17)	34 439	5415	12 554	623/1657 (38%)	15	119	39·5	314 (230–430)	7	56	18·4	147 (93–232)
<15 years	NA	53 747	8443	19 574	NA	30	153	76·1	389 (310–486)	59	301	145·3	742 (631–873)
≥15 years	NA	NA†	NA	NA	NA	NA†	NA	NA	NA	NA†	NA	NA	NA
All	NA	NA†	NA	NA	NA	NA†	NA	NA	NA	NA†	NA	NA	NA
**Pikine, Senegal**‡§													
0–1 years	39% (32–46)	20 120	7837	11 194	NA	0	0	NA	NA	0	0	NA	NA
2–4 years	37% (33–41)	30 180	11 097	15 851	NA	0	0	NA	NA	0	0	..	NA
5–14 years	31% (28–34)	96 152	29 807	42 577	NA	3	7	NA	NA	1	5	..	NA
<15 years	NA	146 452	48 741	69 623	NA	3	4	NA	NA	0	0	..	NA
≥15 years	30% (28–31)	195 726	58 718	83 874	NA	4	5	NA	NA	3	6	..	NA
All	NA	342 178	107 459	153 496	NA	7	5	NA	NA	4	5	..	NA
**East Wad Medani, Sudan**§													
0–1 years	23% (14–32)	2377	537	589	2/85 (2%)	0	0	0·0	0 (0–0)	0	0	0·0	0 (0–0)
2–4 years	22% (15–29)	3566	781	857	29/108 (27%)	0	0	0·0	0 (0–0)	0	0	0·0	0 (0–0)
5–14 years	25% (21–28)	11 071	2735	2999	160/234 (68%)	0	0	0·0	0 (0–0)	0	0	0·0	0 (0–0)
<15 years	NA	17 014	4053	4445	NA	0	0	0·0	0 (0–0)	0	0	0·0	0 (0–0)
≥15 years	29% (27–31)	29 843	8684	9525	131/147 (89%)	0	0	0·0	0 (0–0)	0	0	0·0	0 (0–0)
All	NA	46 857	12 737	13 970	NA	0	0	0·0	0 (0–0)	0	0	0·0	0 (0–0)
**Butajira, Ethiopia**§													
0–1 years	69% (59–78)	2266	1563	2798	NA	0	0	NA	NA	0	0	NA	NA
2–4 years	62% (55–69)	3398	2107	3771	NA	0	0	NA	NA	0	0	NA	NA
5–14 years	65% (61–69)	14 015	9110	16 305	NA	1	6	NA	NA	0	0	NA	NA
<15 years	NA	19 679	12 780	22 874	NA	1	4	NA	NA	0	0	NA	NA
≥15 years	65% (62–68)	42 545	28 080	50 257	NA	2	4	NA	NA	0	0	NA	NA
All	NA	62 224	40 860	73 131	NA	3	4	NA	NA	0	0	NA	NA
**Moshi Rural District, Tanzania**													
0–1 years	4% (0–11)¶	24 289	390	693	79%*	0	0	0·0	0 (0–0)	0	0	0·0	0 (0–0)
2–4 years	2% (0–4)‖	25 281	406	721	79%*	0	0	0·0	0 (0–0)	0	0	0·0	0 (0–0)
5–14 years	13% (10–16)	118 219	15 487	27 508	79%*	2 (4)**	15	5·1	18 (8–44)	0	0	0·0	0 (0–0)
<15 years	NA	167 789	16 283	28 922	NA	2 (4)**	14	5·1	18 (7–42)	0	0	0·0	0 (0–0)
≥15 years	2% (1–2)	298 948	5172	9186	79%*	1 (2)**	22	2·5	28 (8–95)	1 (2)**	22	2·5	28 (8–95)
All	NA	466 737	21 454	38 108	NA	3 (6)**	16	7·6	20 (10–41)	1 (2)**	5	2·5	7 (2–23)
**Moshi Urban District, Tanzania**													
0–1 years	7% (0–19)¶	10 406	335	595	79%*	0	0	0·0	0 (0–0)	1 (2)**	336	2·5	427 (125–1461)
2–4 years	2% (0–6)‖	10 831	348	618	79%*	1 (5)**	809	6·4	1028 (472–2237)	0	0	0·0	0 (0–0)
5–14 years	13% (8–19)	37 309	4850	8615	79%*	2 (7)**	81	8·9	103 (54–199)	0	0	0·0	0 (0–0)
<15 years	NA	58 546	5533	9828	NA	3 (12)**	122	15·2	155 (94–256)	1 (2)**	20	2·5	26 (8–88)
≥15 years	2% (0–3)	125 746	2138	3796	79%*	3 (6)**	158	7·6	201 (99–408)	0	0	0·0	0 (0–0)
All	NA	184 292	7671	13 626	NA	6 (18)**	132	22·9	168 (111–253)	1 (2)**	15	2·5	19 (5–64)
**Kibera, Kenya**††													
0–1 years	42% (38–47)	3467	1456	2031	99/99 (100%)	3	148	3·0	148 (48–458)	1	49	1·0	49 (7–350)
2–4 years	39% (36–43)	3070	1197	2039	312/312 (100%)	10	490	10·0	490 (264–912)	1	49	1·0	49 (7–348)
5–14 years	43% (39–47)	7514	3231	5722	539/539 (100%)	28	489	28·0	489 (338–709)	1	17	1·0	17 (2–124)
<15 years	NA	14 051	5884	9792	NA	41	419	41·0	419 (308–569)	3	31	3·0	31 (10–95)
≥15 years	35% (32–38)	15 263	5342	9228	301/301 (100%)	13	141	13·0	141 (82–243)	3	33	3·0	33 (10–101)
All	NA	29 314	11 227	19 020	NA	54	284	54·0	284 (217–371)	6	32	6·0	32 (14–70)
**Imerintsiatosika, Madagascar**													
0–1 years	28% (20–37)	3424	753	1287	66/85(78%)	0	0	0·0	0 (0–0)	1	78	1·3	100 (18–562)
2–4 years	19% (14–25)	5136	1130	1932	87/101 (86%)	0	0	0·0	0 (0–0)	0	0	0·0	0 (0–0)
5–14 years	18% (15–20)	13 188	2374	4057	184/256 (72%)	5	123	6·9	171 (81–360)	0	0	0·0	0 (0–0)
<15 years	NA	21 748	4257	7276	NA	5	69	6·9	95 (45–201)	1	14	1·3	18 (3–99)
≥15 years	17% (15–19)	24 632	4187	7153	639/919 (70%)	1	14	1·4	20 (4–103)	0	0	0·0	0 (0–0)
All	NA	46 380	8444	14 429	NA	6	42	8·4	58 (29–114)	1	7	1·3	9 (2–50)
**Isotry, Madagasar**													
0–1 years	6% (1–12)	3204	192	261	12/14 (86%)	0	0	0·0	0 (0–0)	0	0	0·0	0 (0–0)
2–4 years	10% (5–14)	4805	481	653	58/65 (89%)	0	0	0·0	0 (0–0)	0	0	0·0	0 (0–0)
5–14 years	9% (7–11)	16 386	1475	2005	234/288 (81%)	1	50	1·2	62 (11–359)	0	0	0·0	0 (0–0)
<15 years	NA	24 395	2147	2919	NA	1	34	1·2	42 (7–247)	0	0	0·0	0 (0–0)
≥15 years	9% (7–11)	45 928	4134	5621	1197/1421 (84%)	2	36	2·4	42 (12–151)	0	0	0·0	0 (0–0)
All	NA	70 323	6281	8540	NA	3	35	3·6	42 (15–119)	0	0	0·0	0 (0–0)
**Pietermaritzburg, South Africa**§													
0–1 years	11% (5–17)	13 990	1511	3055	NA	0	0	NA	NA	0	0	NA	NA
2–4 years	7% (3–12)	20 985	1490	3013	NA	0	0	NA	NA	0	0	NA	NA
5–14 years	16% (13–19)	62 313	10 157	20 537	NA	0	0	NA	NA	0	0	NA	NA
<15 years	NA	97 288	13 158	26 605	NA	0	0	NA	NA	0	0	NA	NA
≥15 years	15% (13–17)	294 542	43 887	88 739	NA	2	2	NA	NA	0	0	NA	NA
All	NA	391 830	57 045	115 344	NA	2	2	NA	NA	0	0	NA	NA

Study population was adjusted for health-seeking behaviour and crude cases were adjusted for recruitment proportion (number of patients analysed divided by number of patients with febrile illness from study area who visited a recruitment health facility, multiplied by 100). iNTS=invasive non-typhoidal salmonella. NA=not available. PYO=person-years of observation.

* *Recruitment portion was not available for each age strata. Broader values were applied to each stratum.

† Target population for surveillance activities in Ghana included patients younger than 15 years of age; patients aged 15 years or older were not recruited.

‡ Three Salmonella Paratyphi A were identified at this site, but are not included in this table.

§ No salmonella was isolated in Sudan. Missing data on recruitment patterns in Senegal, Ethiopia, and South Africa did not allow calculation of adjusted incidences. Crude rates are presented.

¶ This proportion applies to age group <1 year, and it was used to adjust the study population by health-seeking behaviour.

‖ This proportion applies to age group 1–4 years, and it was used to adjust the study population by health-seeking behaviour. The adjusted populations in age groups <1 year and 1–4 years were added to estimate the total adjusted population age group 0–4 years. Subsequently, the percentage of children <2 years reported by the 2012 national census was applied to derive age groups 0–1 years and 2–4 years.

** Crude cases have been adjusted for recruitment pattern unique to the site in Tanzania: before Nov 11, 2011, every fifth eligible patient was recruited; from Nov 11, 2011, every second eligible patient was recruited. Adjusted cases (presented inside parentheses) were used to calculate crude rate.

†† Active population mobilisation was done, in addition to passive surveillance.

**Table 3:** Antimicrobial resistance patterns of *Salmonella enterica* serotype Typhi and iNTS isolates across sites

		**Burkina Faso**	**Guinea-Bissau**	Senegal*	**Ghana**	**Ethiopia**	**Madagascar**	**South Africa**	**Tanzania**	**Kenya**	**All**
Total *S* Typhi isolates, N		18	3	7	30	3	9	2	9	54	135
Isolate with antimicrobial resistance, n (%)†											
	Ampicillin	0	NR	NR	20 (67%)	2 (67%)	NR	0	8 (89%)	41 (76%)	71 (53%)
	Amoxicillin-clavulanic acid	0	NR	NR	3 (10%)	0	NR	0	4 (44%)	24 (44%)	31 (23%)
	Chloramphenicol	2 (11%)	NR	NR	23 (77%)	0	NR	0	5 (56%)	43 (80%)	73 (54%)
	Co-trimoxazole	2 (11%)	NR	NR	24 (80%)	0	NR	0	8 (89%)	43 (80%)	77 (57%)
	Ceftriaxone	0	NR	NR	0	0	NR	0	0	0	0
	Ciprofloxacin	0	NR	NR	0	0	NR	1 (50%)	0	11 (20%)	12 (9%)
	Multidrug resistance‡	0	NR	NR	19 (63%)	0	NR	0	5 (56%)	40 (74%)	64 (47%)
	Total iNTS isolates, N	14	8	4	59	0	1	0	2	6	94
Isolate with antimicrobial resistance, n (%)†											
	Ampicillin	10 (71%)	1 (13%)	NR	38 (64%)	NR	NR	NR	0	2 (33%)	51 (54%)
	Amoxicillin-clavulanic acid	3 (21%)	0	NR	9 (15%)	NR	NR	NR	0	2 (33%)	14 (15%)
	Chloramphenicol	12 (86%)	1 (13%)	NR	34 (58%)	NR	NR	NR	0	1 (17%)	48 (51%)
	Co-trimoxazole	13 (93%)	1 (13%)	NR	34 (58%)	NR	NR	NR	0	2 (33%)	50 (53%)
	Ceftriaxone	0	0	NR	0	NR	NR	NR	0	1 (17%)	1 (1%)
	Ciprofloxacin	1 (7%)	0	NR	2 (3%)	NR	NR	NR	0	0	3 (3%)
	Multidrug resistance‡	10 (71%)	1 (13%)	NR	33 (56%)	NR	NR	NR	0	1 (17%)	45 (48%)

Resistant isolates are reported per country, rather than per site. No *Salmonella enterica* serotype Typhi (*S* Typhi) or iNTS isolates were cultured in Sudan. iNTS=invasive non-typhoidal salmonella. NR=no resistant isolates identified.

* Seven *S* Typhi, four iNTS, and three *S enterica* serotype Paratyphi (*S* Paratyphi) isolates. One of the *S* Paratyphi isolates was resistant to ciprofloxacin.

† Includes isolates fully and intermediately resistant against the respective drug, as defined by the Clinical Laboratory and Standards Institute guidelines 2013.^15^

‡ Defined as resistance against ampicillin or amoxicillin AND chloramphenicol AND co-trimoxazole.
